# A method to determine the displacement velocity field in the apical region of the Arabidopsis root

**DOI:** 10.1007/s00425-012-1707-x

**Published:** 2012-07-25

**Authors:** Jerzy Nakielski, Marcin Lipowczan

**Affiliations:** Department of Biophysics and Morphogenesis of Plants, University of Silesia, Katowice, Poland

**Keywords:** Arabidopsis root apex, Displacement velocities, Mathematical modelling, Velocity field

## Abstract

In angiosperms, growth of the root apex is determined by the quiescent centre. All tissues of the root proper and the root cap are derived from initial cells that surround this zone. The diversity of cell lineages originated from these initials suggests an interesting variation of the displacement velocity within the root apex. However, little is known about this variation, especially in the most apical region including the root cap. This paper shows a method of determination of velocity field for this region taking the Arabidopsis root apex as example. Assuming the symplastic growth without a rotation around the root axis, the method combines mathematical modelling and two types of empirical data: the published velocity profile along the root axis above the quiescent centre, and dimensions of cell packet originated from the initials of epidermis and lateral root cap. The velocities, calculated for points of the axial section, vary in length and direction. Their length increases with distance from the quiescent centre, in the root cap at least twice slower than in the root proper, if points at similar distance from the quiescent centre are compared. The vector orientation depends on the position of a calculation point, the widest range of angular changes, reaching almost 90°, in the lateral root cap. It is demonstrated how the velocity field is related to both distribution of growth rates and growth-resulted deformation of the cell wall system. Also changes in the field due to cell pattern asymmetry and differences in slope of the velocity profile are modelled.

**Electronic supplementary material:**

The online version of this article (doi:10.1007/s00425-012-1707-x) contains supplementary material, which is available to authorized users.

## Introduction

Cells of plant organs typically grow in a coordinated manner, i.e., symplastically (Erickson 1986). During symplastic growth, when definable points of the organ displace relative to some reference point, contiguous walls of neighbouring cells do not slide or slip with respect to each other preserving their mutual contacts (Priestley 1930; Romberger et al. 2004). A displacement versus time defines the displacement velocity (**V**) at a given point (Silk 1984). If the velocity is determined at every point, there is a vector field of the displacement velocity within the organ (Hejnowicz 1982; Gandar and Chalabi 1989). The coordination extended on the whole cell wall system means that **V** field at the organ level is continuous. In this sense, though the organ is complex and not uniform in structure, its growth can be considered as continuous. Manifestations of this continuity are observed in cell packets formed within and at the surface of the growing organ (Nakielski 1987; Hejnowicz et al. 1988; Kwiatkowska 2004).

Mathematically, the displacement velocity field is represented by continuous and differentiable function of position (Hejnowicz 1982; Silk 1984; Gandar and Chalabi 1989). This function defines velocities with all definable points of the organ relative to some reference point. If **V** is known, there are two approaches to growth analysis. One, via differentiation of **V**, leads to determination of a spatial variation of growth rates within and at the surface of the organ (Nakielski 1991; Hejnowicz and Karczewski 1993). The other, via integration of **V**, allows one to calculate growth-resulting deformations of the organ (Hejnowicz 1989; Kennaway et al. 2011) and, using a simulation models, to generate growth with cell divisions (Nakielski 2008; Szymanowska-Pułka and Nakielski 2010). Both ways are important for an overall description of the growth and better understanding of underlying biophysical mechanisms (Hejnowicz and Romberger 1984; Nakielski and Hejnowicz 2003).

Despite their importance, velocity fields in growing plant organs are poorly known. In the case of root apices, our knowledge is mostly limited to a profile showing how the displacement velocity changes along the root axis with increasing distance from the tip. Such velocity profiles were obtained on the basis of streak photography, marking experiments and anatomical records (Erickson and Sax 1956; Silk et al. 1989), and by advanced methods of kinematic analysis applied to in vivo confocal scanning microscopy images (Van der Weele et al. 2003; Basu et al. 2007; Chavarria-Krauser and Schurr 2004; Roberts et al. 2010). The results include valuable information about **V**, but usually restricted to one direction, along the root axis. Recently, these velocities have been determined also away from the axis (Wuyts et al. 2011), but not for the most apical part of the organ that is especially interesting from the growth organisation perspective (Dolan et al. 1993; Jiang and Feldman 2005). This motivates a search for further methods.

In the apical region of angiosperms roots, the quiescent centre (Clowes 1956), i.e., the zone of a low mitotic activity, acts as a kind of an organizational centre that determines growth and cell pattern of the root apex (Webster and MacLeod 1996; Jiang and Feldman 2005). All tissues of the root proper and the root cap are derived from initial cells surrounding the quiescent centre. In the case of *Arabidopisis thaliana* root apex, which exhibits a relatively simple cellular organization, initials of particular tissues have been precisely recognized (Dolan et al. 1993). We know their location within the meristem and how they and their derivatives divide. A diversity of cell lineages originating from the initials, observed in the course of intact growth and as a result of laser ablation experiments (Van den Berg et al. 1995; Kidner et al. 2000; Scheres et al. 2002), suggests that there exists an interesting spatial and directional variation of **V** vectors in most apical region of the root. What are the velocities in this region, in different parts of the root proper and the root cap?

The present paper aims at answering this question. Assuming the symplastic growth and continuity of **V**, the method of determination of the displacement velocity for an *A. thaliana* root apex is described. The method combines mathematical modelling and two types of empirical data: the published velocity profile along the root axis above quiescent centre (Van der Weele et al. 2003) and, using cell pattern in the axial section, dimensions of cell packet derived from the initials of epidermis and lateral root cap. The results demonstrate the **V** field for apical region including the root cap. Results are also shown in conjunction with distribution of growth rates and growth-resulted deformation of cell wall system. In addition, changes in the **V** field due to cell pattern asymmetry and differences in slope of the velocity profile are modelled.

## Materials and methods

The *A. thaliana* L. root apex of about 1-week-old seedlings was modelled. Geometry and cell pattern of the apex up to 120 μm from the summit (axial section) were adopted from published papers (Van den Berg et al. 1997; Scheres et al. 2002; Campilho et al. 2006) in which they are regarded as typical for the wild type. The cell pattern was digitized and arranged into a meshwork in which individual cells were described by polygons (Nakielski 2008). The meshwork represented the root apex in modelling and computer simulations, as input data.

**Fig. 1:**
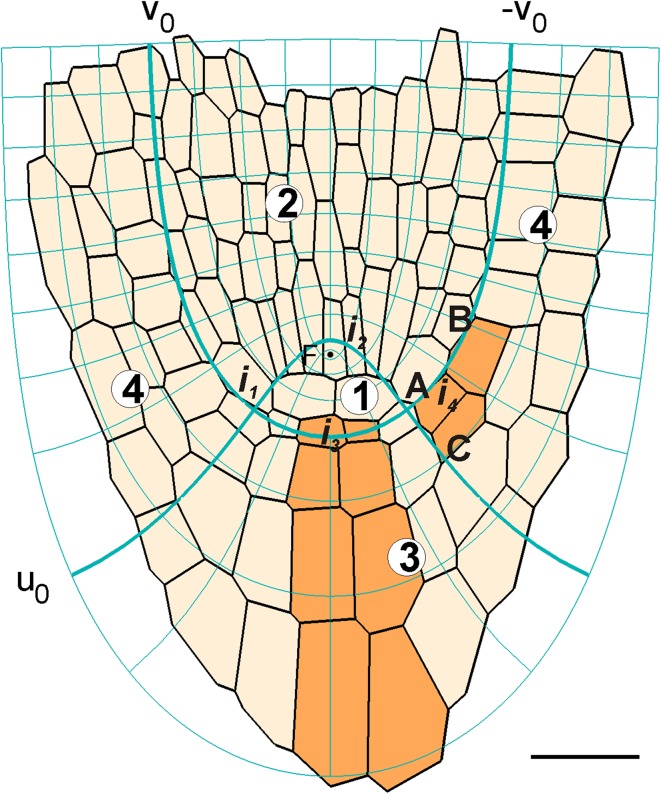
The cell pattern of *A. thaliana* root apex (digitized on the basis of axial section shown in Van den Berg et al. 1997) with the applied root-designated coordinate system, RC (*u*, *v*, *φ*), taken for *φ* = const. The system (*thin blue*) is curvilinear, orthogonal and of confocal type. Two coordinate lines: *u*_0_ and *v*_0_ turning into −*v*_*0*_ (*thick blue*), divide the apex into four zones corresponding to: zones 1, 2—the root proper without epidermis; zones 3, 4—the root cap with epidermis, the zone 1 including the focus (F) represents the quiescent centre. The exemplary initials of particular tissues are indicated: *i*_*1*_, initial of cortex and endodermis; *i*_*2*_, vascular cylinder; *i*_*3*_, columella; *i*_*4*_, epidermis and lateral part of the root cap. The distinguished cell packets are used to specify components of the displacement velocity in this paper; AB and AC are dimensions of the cell packet derived from *i*_*4*_ along *v*_0_ and *u*_0_, respectively. *Bar* = 20 μm

In order to describe geometry and displacement velocities in points of the apex, the root-designated coordinate system (Hejnowicz and Karczewski 1993) was used. The system, here referred to as RC (*u*, *v*, *φ*), is curvilinear orthogonal and of confocal type. For *φ* = const which corresponds to the axial plane, there are two families of mutually orthogonal lines *u* = const and *v* = const (Fig. 1; Online Resource S1). The focus *F* located at the root axis divides this axis into two parts which are represented by *v* = 0 above, and *u* = 0 below *F*. An application of the system to cell pattern of the apex was such that taking *v*_*0*_ = π/4 which turns into −*v*_*0*_ = −π/4 as the border between the root proper and the root cap (Fig. 1), the line *u*_0_ = 0.35 represented the basal limit of the quiescent centre and the border between the columella and lateral part of the root cap. It was assumed that under such application, the root apex grows steadily without a rotation around the root axis.

Let us take the focus *F* as the reference point. In general, the **V** vector in RC (*u*, *v* ,*φ*) system is composed of three physical components *V*_*u*_, *V*_*v*_, and *V*_φ_. Due to absence of a rotation, the displacement velocity consists of two components: basipetal and acropetal that are responsible for displacements of cells towards the basal and apical part of the organ, respectively. The basipetal component of **V** was specified by the velocity profile along the root axis above the quiescent centre adopted from Van der Weele et al. (2003). This profile, obtained using advanced computational image analysis of deformable motion of the root apex at high spatial and temporal resolution, is composed of three distinct regions: the velocity increases linearly with distance from the quiescent centre, first gradually, then steeply, and finally it becomes constant at the end of the growth zone. In correspondence to the assumed cell pattern, data from the first region, at the segment up to 120 μm from the summit, were taken into account. According to the data, the velocity increased 1.5 μm per minute, per every millimeter of the distance from the quiescent centre.

The acropetal component of **V** was specified by dimensions of the cell packet originated from the initial, common for the rhizodermis and lateral part of the root cap (*i*_4_ in Fig. 1). These dimensions, expressed by the ratio of the length along *u*_0_ (AC) to the length along *v*_0_ (AB) gave the values: AC/AB = 0.6 for the right, and AC/AB = 0.54 for the left side of the root apex. Also cell packets in several other published root apices were measured, obtained values of the ratio were in the range from AC/AB = 0.60 (Kurup et al. 2005) to AC/AB = 0.80 (Friml 2003).

The other qualitative data came from the cell lineage derived from the columella initial (*i*_3_ in Fig. 1). In the lineage, length of cells along the root axis was measured. For every two consecutive cells within the lineage ratios of their lengths were the following: *l*_1_/*l*_ini_ = 2.40, *l*_2_/*l*_1_ = 1.78, *l*_3_/*l*_2_ = 1.34, where *l*_ini_ is the length of the initial. Values of the ratios estimated for several other published roots apices (Dolan et al. 1993; Rost et al. 1996; Friml 2003; Sarkar et al. 2007) gave the following results: *l*_1_/*l*_ini_ = 1.80, *l*_2_/*l*_1_ = 1.69, *l*_3_/*l*_2_ = 1.33, and *l*_4_/*l*_3_ = 1.21 when four derivatives in the lineages occurred.

For specification of **V** components, the 2D simulation model for growth and cell division was used (Nakielski 2008). The model is composed of three elements: the polygon meshwork representing cell pattern at the input, growth field generating growth, and the algorithm including rules for cell divisions. The temporal sequences of the simulated growth are obtained by operational application of the growth field to the meshwork. During the simulated growth, the meshwork expands, deforms, and new cells are formed through divisions. The division can be longitudinal or transversal. Both orientations of a division wall are considered and finally, the direction for which the division wall can be shorter is chosen. After formation, the division wall is shortened slightly with respect to its former length. This results in three-way junctions at new vertices similar to that ones usually occurring in a real organ, and the simulated growth is continued.

In our application, the growth field resulted from differentiation of **V** and the model worked in two modes: with and without cell divisions. The divisions were included in simulation of development of the columella cell files, whereas they were excluded in testing of growth-resulting deformation of the tetragon assumed to describe cell packet formed in the epidermis and lateral root cap.

The **V** field was presented in a form of vectors drawn for selected points of the axial plane. The other figures demonstrated how the **V** field is related to distribution of the volumetric growth rate and growth-resulting deformation of cell wall system in the whole root apex. For the rate distribution, values of the relative elemental rate of the volumetric growth (*R*_vol_) were calculated as the divergence of **V** (Hejnowicz 1982). These values, attributed to cells from Fig. 1, represented the cell centres. The cell walls deformation was obtained using the above mentioned 2D simulation model for growth (Nakielski 2008) in the mode in which cell divisions were excluded. New positions of particular cells were calculated from old ones by integration of components of **V** with respect to time.

## Results

### Displacement velocities in RC (*u*, *v*, *φ*) system

**Fig. 2:**
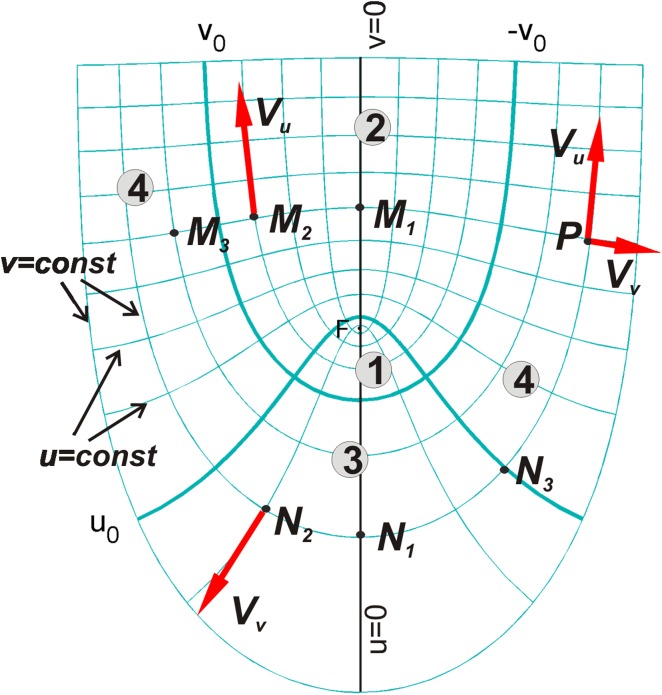
The components of the displacement velocity in the RC (*u*, *v*, *φ*) system. Under absence of rotation, the **V** consists of *V*_*u*_ = *h*_*u*_ *du*/*dt* and *V*_*v*_ = *h*_*v*_ *dv*/*dt* where *h*_*u*_, *h*_*v*_ are the scale factors. Both components are zero in the quiescent centre and nonzero in the lateral root cap with epidermis. In two remaining zones there are nonzero *V*_*u*_ in stele, and *V*_*v*_ in the central root cap. Because *h*_*u*_ and *h*_*v*_ change with position, points lying at a common line *u* = const, as M_1_, M_2_, M_3_, P, have the same d*u*/d*t* but different *V*_*u*_, and similarly, points lying at the common line *v* = const that turns into −*v* = const, as N_1_, N_2_, N_3_, P, have the same d*v*/d*t* but different *V*_*v*_. On the basis of this property the knowledge of *V*_*v*_ along one of *u* = const lines, in particular *u* = 0 (part of the root axis below F), and *V*_*u*_ along one of *v* = const lines, in particular *v* = 0 (part of the root axis above F), is sufficient to determine **V** at any point of the organ

Under the assumed application of the RC (*u*, *v*, *φ*) system to cell pattern (Fig. 1) two lines: *v*_0_ which turns into −*v*_0_, and *u*_0_ define four zones of the root apex representing: zone 1, the quiescent centre with parts of surrounding initials; zone 2, stele; zone 3, central root cap; zone 4, lateral root cap and epidermis. For the axial plane and the absence of a rotation, we have (Fig. 2): *V*_*u*_ = *h*_*u*_ d*u*/d*t*, *V*_*v*_ = *h*_*v*_ d*v*/d*t* and *V*_φ_ = 0 where *h*_*u*_ and *h*_*v*_ are scale factors of the system (see Online Resource S1). As in previous modelling (Hejnowicz and Karczewski 1993; Nakielski 2008), it is assumed that: d*u*/d*t* = 0 and d*v*/d*t* = 0 in the quiescent centre with parts of surrounding initials; d*u*/d*t* = *c*(*u* − *u*_*0*_) and d*v*/d*t* = 0 in stele; d*u*/d*t* = 0 and d*v*/d*t* = −*d*sin (*qv*) in the central root cap; d*u*/d*t* = *c*(*u* − *u*_*0*_), and d*v*/d*t* = −*d*sin(*qv*) in the lateral root cap and epidermis, where *q* = π/*v*_0_, and *c*, *d* are constants. According to these equations, cells located in the quiescent centre do not grow at all, whereas the remaining cells grow and displace away from the quiescent centre (Fig. 2): basipetally along *v* = const in stele, acropetally along the *u* = const in the central root cap, and towards the root periphery in the direction depending on the ratio *V*_*u*_/*V*_*v*_ in the lateral root cap and epidermis. Notice that such defined **V** field is continuous and, if both scale factors of the coordinate system are known (see Online Resource S1), *V*_*u*_ depends only on d*u*/d*t* determined by the parameter *c*, whereas *V*_*v*_ depends only on d*v*/d*t* determined by the parameter *d*. It means that in order to determine *V*_*u*_ and *V*_*v*_, we need to specify *c* and *d*, respectively.

The continuity of **V** field implies interesting properties related to *V*_*u*_ and *V*_*v*_ at different points of the growing organ (Hejnowicz 1982, 1984). Namely, points lying on a common line *u* = const (Fig. 2) have the same d*u*/d*t* though they differ from each other concerning *V*_*u*_ (because of *h*_*u*_ which changes with *v*). Similarly, points lying on a common line *v* = const have the same d*v*/d*t* though they differ from each other concerning *V*_*v*_ (because of *h*_*v*_ which changes with *u*). It means that the knowledge of *V*_*u*_ along one of *v* = const lines, and *V*_*v*_ along one of *u* = const lines, is sufficient to determine both components, *V*_*u*_ and *V*_*v*_, at any point of the root apex. Applying these properties, the parameters *c* and *d* can be specified particularly for the root axis, at the segments above (along *v* = 0) and below (along *u* = 0) the focus, respectively. Such specification is sufficient to reconstruct **V** field for the whole root apex.

### Specification of components of **V** by empirical data

**Fig. 3:**
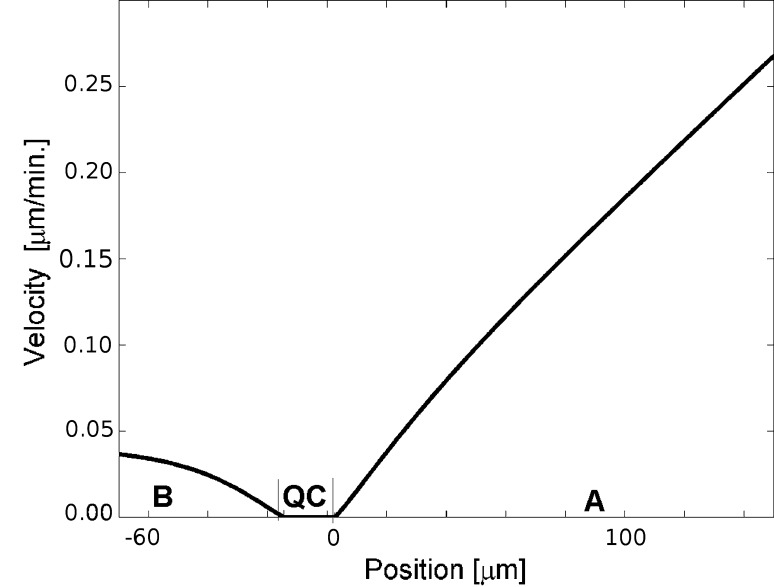
The displacement velocity profiles taken to specify *V*_*u*_ and *V*_*v*_ in this paper. Both components increase with the distance from the quiescent centre, the *V*_*u*_ basipetally with *c* = *0.*8 (part A), the *V*_*v*_ acropetally with *d* = 0.12 (part B). At the segment corresponding to the quiescent centre there is no growth at all

**Fig. 4:**
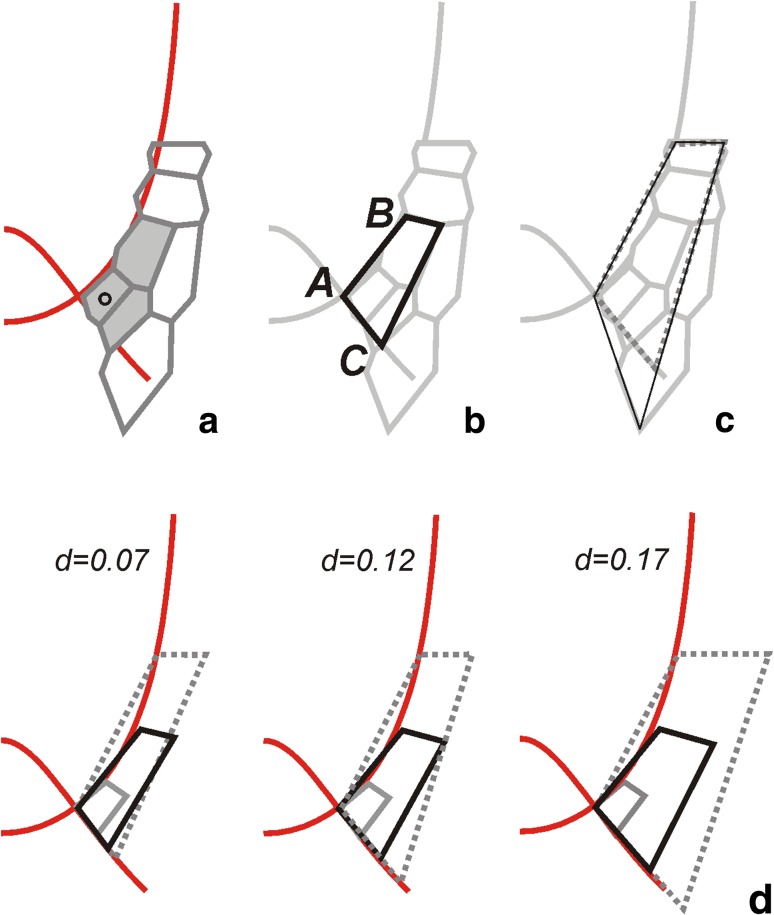
The method of *V*_*v*_ specification. **a** The cell packet that derived from the initial of rhizodermis and lateral part of the root cap; from this initial (*circle*) at first the cell triad (*grey*), then the whole packet are formed; the zones are as in Fig. 1, *red lines* mark *u*_0_ and *v*_0_. **b** The tetragon (*black*) describing the part of the cell packet localized in the lateral root cap and epidermis at the stage of the cell triad: AB, the length along *v*_0_; AC, length along *u*_0_. **c** The tetragons assumed to describe the whole cell packet (*thin black*) and the region of the cell packet localized in the lateral root cap and epidermis (*grey dashed*), i.e., without the part protruding from this zone. **d** The tetragons of different AC/AB ratios obtained in the course of the computer simulation at times corresponding to three different stages of the cell packet development. Assuming different values of the parameter *d* for constant AB determined by *c* = 0.8 the most realistic shapes corresponding to *black tetragon* in **b** and *grey dashed tetragon* in **c** are achieved for *d* = *0.*12. For more explanation, see text

As mentioned earlier, the velocity profile along the root axis at the segment up to 120 μm from the tip, adopted from Van der Weele et al. (2003), was assumed to specify *V*_*u*_. The profile was described using the linear function d*u*/d*t* = *cu.* Value of the parameter *c* was determined by measurement of the slope of the profile on the basis of Fig. 3 of the quoted paper. The best approximation of this profile, according to which velocity increases about 1.5 μm per minute, per every millimeter of the distance from the quiescent centre, has been achieved for *c* = 0.8. This gave *V*_*u*_ (*u*, 0) = 0.8*u* for the part A (Fig. 3) of the root axis. The obtained values of the parameter *c* resulted in an almost constant growth rate similar as demonstrated in Fig. 4 of the source paper (Van der Weele et al. 2003). The values of *V*_*u*_ away from the root axis were calculated from the equation (Hejnowicz 1982, 1984):

$$ {V_{u} (u,v) = \frac{h_u(u,v)}{h_u(u,0)}V_{u} (u,0)} $$

The component *V*_*v*_ for the part B of the root axis that includes the root cap (Fig. 3) was specified by another type of data, namely the data coming from observation of cell pattern in the lateral root cap and epidermis. Note that in this region *V*_*u*_ and *V*_*v*_ coexist (Fig. 2) and accordingly, both these components are manifested in cell packets formed within this region. Let us consider the cell packet originating from the initial which is common for the rhizodermis and lateral part of the root cap. Being initiated in the corner of this zone (Fig. 4a), this cell packet is exceptionally useful for our specification because its dimensions can be relatively easily measured along the lines *u*_0_ and *v*_0_ representing borders of this zone. Moreover, the cell packet length along *v*_0_ depends only on *c*, whereas along *u*_0_ only on *d*, in terms of our approach. Let us focus our attention on the stage when the cell packet consists of three cells (Fig. 4b) and note a part of the cell triad situated between the lines *u*_0_ and *v*_0_. Assuming the shape of this part as tetragonal, its dimensions are represented by AC along *u*_0_ and AB along *v*_0_. For the considered cell triad, both lengths were measured giving the ratio AC/AB = 0.6.

Knowing the ratio AC/AB and the value of the parameter *c*, specified above, the *d* was estimated using the 2D simulation model of the root growth (Nakielski 2008), which is based on exactly the same assumptions concerning the **V** field as those adopted here. Figure 4d shows computer-generated sequences of development of the tetragon obtained for *c* = 0.8 under different *d* values in the range from *d* = 0.07 to *d* = 0.17. Such the range includes the ratio AC/AB obtained for cell packets originated from the same initial on the left side of the considered root apex as well as mentioned extreme values of this ratio found in literature. Among three virtual tetragons drawn in Fig. 4d by black lines, the most realistic shape, i.e., the most similar to corresponding tetragon in Fig. 4b describing the real cell packet but only in the part limited to the lateral root cap and epidermis (zone 4 in Fig. 4), has been achieved for *d* = 0.12. The other two cases gave the AC segment either too short (*d* = 0.07) or too long (*d* = 0.17) in comparison with Fig. 4b. Moreover, if *d* = 0.12 is assumed, also the shape obtained at next time looks realistically (grey dotted tetragon in Fig. 4d resembles the dotted tetragon in Fig. 4c). Such *d* specification provided the profile of *V*_*v*_ along *u* = 0 as shown in Fig. 3 (part B) giving *V*_*v*_ (*0*, *v*) = 0.12 d*v*/d*t*. The values of *V*_*v*_ away from the root axis were calculated from the equation (Hejnowicz 1982, 1984):

$$ {V_v\,(u,v) = \frac{h_v\,(u,v)}{h_v\,(0,v)}V_v\,(0,v)} $$

**Fig. 5:**
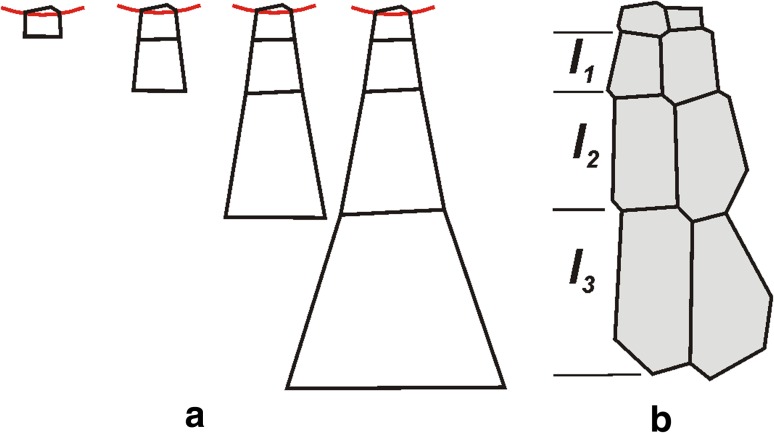
Comparison of the columella cell lineages. **a** Generated by computer for *d* = *0.12*. **b** Observed in real root apex from Fig. 1. Temporal sequence shown in **a** has been obtained using the simulation model for growth and cell divisions (Nakielski 2008); *red line* represent the distal border of the quiescent centre. In both, real and virtual lineages, longitudinal dimensions *l*_1_, *l*_2_, *l*_3_ of corresponding cells are similar

To verify the obtained value of the parameter *d*, the simulations were applied also to the cell packet that develops in the central root cap where only *V*_*v*_ occurs. The simulation model was the same as in the case of the tetragon (Fig. 4d), except for the fact that here cells were able to divide. Assuming *d* = 0.12, successive steps of developing the columella cell lineage originated from the initial located at the quiescent centre border are shown in Fig. 5a. At the final step, longitudinal dimensions *l*_1_, *l*_2_, *l*_3_ of successive cells in the lineage, as well as the ratios: *l*_1_/*l*_ini_, *l*_2_/*l*_1_, *l*_3_/*l*_2_ were similar to observed in the real root apex (Fig. 5b). It means that the obtained value of the parameter *d* describes well the columella cell lineages of the considered root apex. Moreover, it may be assumed as representing also for other root apices because, excluding the ratio *l*_1_/*l*_ini_ as depending strongly on actual state of the initial cell, the remaining ratios estimated for several other published roots apices are more or less similar to those in the virtual lineage, at least if longitudinal cell dimensions are compared.

The specified values of the parameters *c* and *d*, in the above equations included in *V*_*u*_ (*u,* 0) and *V*_*v*_ (0, *v*), respectively, were constant through particular zones. Nevertheless, *V*_*u*_ (*u, v*) and *V*_*v*_ (*u*, *v*) vary with position as a result of strong dependence of d*u*/d*t* on *u*, d*v*/d*t* on *v*, and scale factors *h*_*u*_, *h*_*v*_ on both *u* and *v* (see Online Resource S1).

### Velocity field in the apical region of Arabidopsis root

**Fig. 6:**
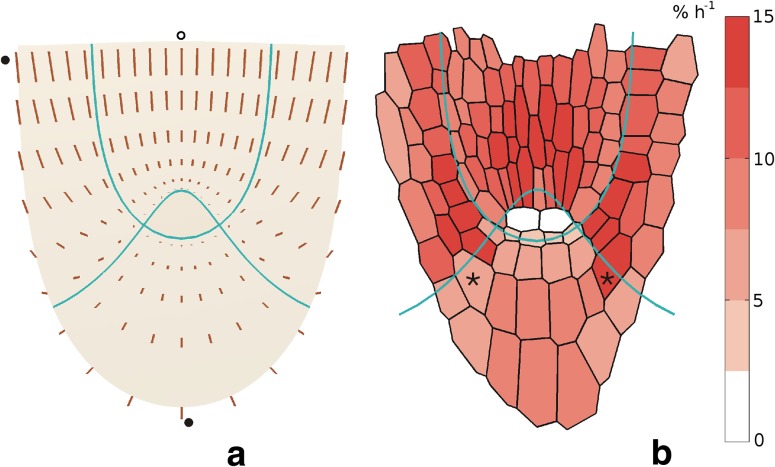
The symmetric velocity field (**a**) and distribution of the volumetric growth rate (**b**) obtained for *A. thaliana* root apex. In **a**, **V** vectors are represented by line segments, the vectors marked by circles correspond to velocities (in μm min^−1^): 0.093 (*closed circle at the top*), 0.039 (*closed at the bottom*) and 0.087 (*open* *circle*). In **b**, values of the rate, colour-coded, are attributed to cells from Fig. 1. In two cells localized in the quiescent centre there is no growth, for two other cells marked by *asterisks*—see text

The displacement velocity field obtained for the *A. thaliana* root apex is shown in Fig. 6a. The **V** vectors vary within the apex in both length and orientation. Their length increases with distance from the quiescent centre and values of **V** in the root cap are at least twice lower than in the root proper, if compared points are lying at similar distance from the quiescent centre. The **V** orientation changes in accordance with our assumption about *V*_*u*_ and *V*_*v*_, i.e., the vectors situated in stele and the central root cap are tangent to the lines *v* = const and *u* = const, respectively, whereas those in the lateral root cap and epidermis manifest orientation depending on the ratio *V*_*u*_/*V*_*v*_ at a given position. In the basal part of the lateral root cap and epidermis, *V*_*u*_ predominates. However, moving along *v* = const with decreasing *u*, this component decreases, and larger and larger participation of *V*_*v*_ can be observed and finally, *V*_*u*_ disappears, whereas *V*_*v*_ reaches maximum at the distal border of this zone.

Figure 6b shows distribution of the volumetric growth rate corresponding to **V**. The *R*_vol_ varies with position in the root apex and the highest values, about 13–14 % h^−1^, are in the innermost part of the zone corresponding to the lateral root cap with epidermis and the central part of stele. Excluding the quiescent centre where there is no growth, the smallest *R*_vol_ values are in the central root cap. Minimum, at the level 2–3 % h^−1^, is in the region adjacent to the quiescent centre. The growth rate distribution, similarly as **V** field, is symmetrical with respect to the root axis, though in Fig. 6b, some differences in *R*_vol_ distribution between both sides of apex occur. Let us take, for example, two cells at similar positions but located on the opposite sides of the root axis (asterisks in Fig. 6b). The difference in their *R*_vol_ values comes mainly from asymmetric distribution of the columella files and the fact that the rates are calculated for points corresponding to cell centres. Simply, centres of these cells happen to different zones, namely the central root cap on the left, and the lateral root cap and epidermis on right side of the root apex.

**Fig. 7:**
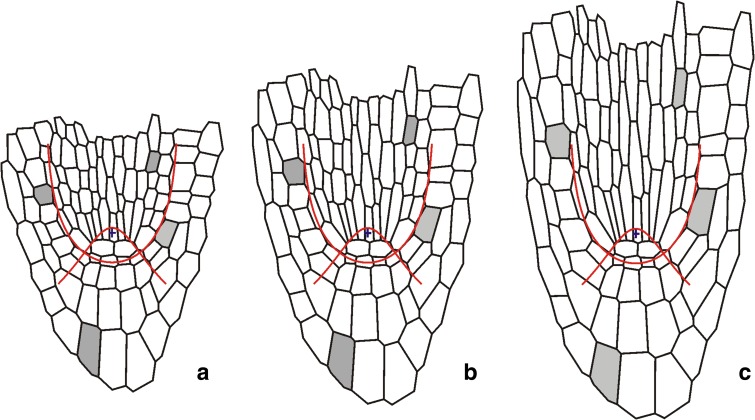
The simulated deformation of the cell wall system from under the **V** field from Fig. 6. The exemplary cells are marked (*grey*), *red lines* show the assumed zones of the root apex

How the **V** field is related to the simulated cell expansion is shown in Fig. 7. Cells of the root proper increase their dimensions faster than those in the root cap and the largest growth-resulting deformations of the cell pattern are in the basal part of the system, in stele and the lateral root cap with epidermis. The smallest deformations occur in the region surrounding QC, especially in the central root cap.

**Fig. 8:**
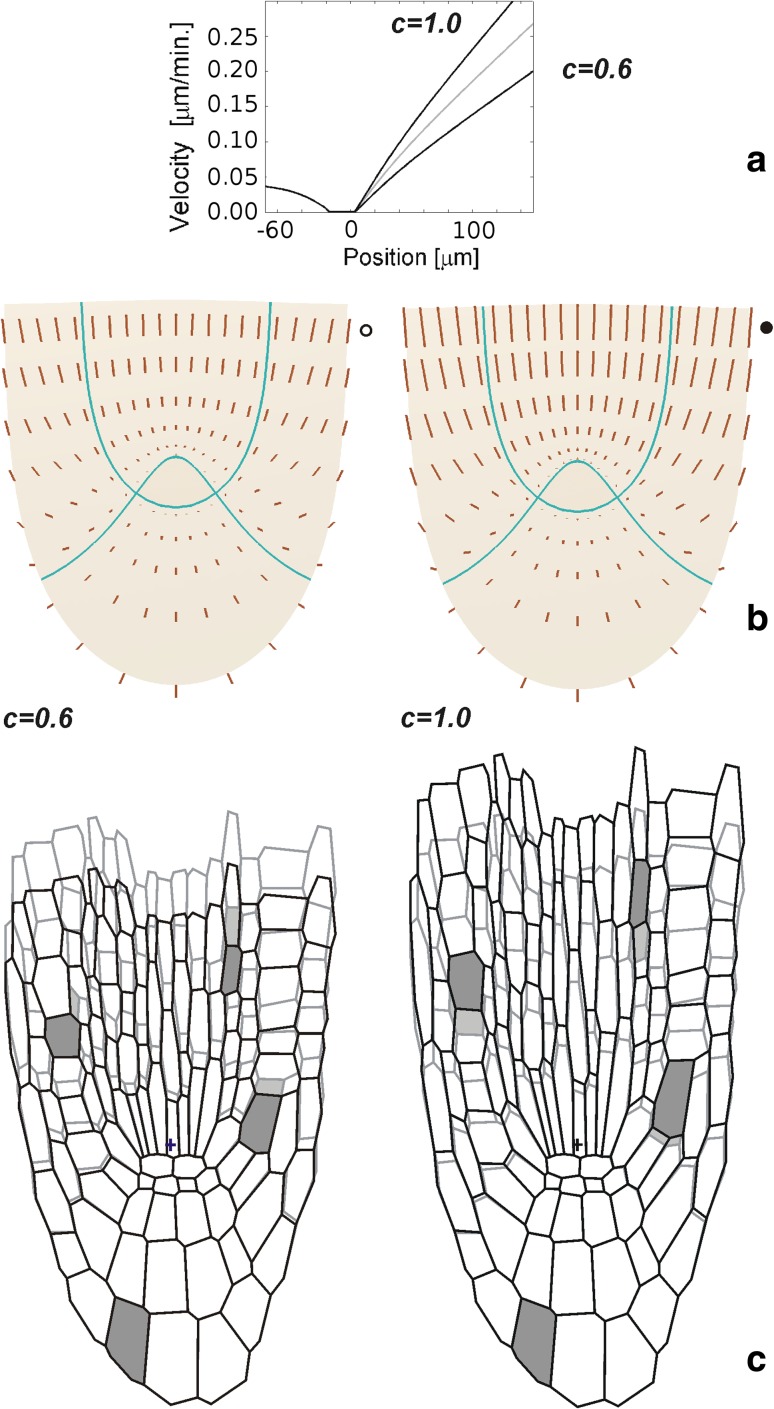
The modification of the velocity profile along the root axis above QC. **a** Two profiles defined for *c* = 0.6 and *c* = 1.0, in both the profile at the segment below QC is specified *d* = 012. **b** **V** fields obtained for these profiles. Values of the marked vectors in units of Fig. 5a are: 0.069 μm min^−1^ (*open circle*) and 0.108 μm min^−1^ (*closed circle*). **c** The growth-resulting deformation of cell wall system under both fields, the walls in the background (*grey*) represent the case *c* = 0.8 from Fig. 7c

The above results have been obtained assuming *c* = 0.8 and *d* = 0.12 that specified *V*_*u*_ and *V*_*v*_, respectively. The question arises how the results change, if slightly larger or smaller values of these parameters modifying slope of the velocity profiles, are assumed. Let us consider the case when the modification is limited to the parameter *c* under unchanged *d* (Fig. 8) that leads to velocity changes in stele (only vector length) and the lateral root cap with epidermis (both length and orientation). For *c* = 0.6, the velocities are lower, whereas for *c* = 1.0, higher in comparison to those in Fig. 6a and, in consequence, there occur less and more pronounced, respectively, differences in **V** between the root proper and the root cap (Fig. 8b). These differences are manifested in deformation of the cell wall system (Fig. 8c); for *c* = 0.6, the increase of dimension of cells in basal peripheries of the system is smaller, whereas for *c* = 1.0, larger than in Fig. 7.

What happens when the parameter *d* is modified under unchanged *c* is addressed in Online Resource S2. Now the changes are observed in the central root cap (only concerning vector length) and lateral root cap (both length and orientation). For *d* = 0.07, the velocities are generally smaller, whereas for *d* = 0.17, greater than those in Fig. 6a which leads, in turn, to more and less pronounced, respectively, difference in **V** values between the root proper and the root cap. The simulated deformation of the cell wall system indicates that increase of cell dimension in the root cap for *d* = 0.07 is smaller, and for *d* = 0.17, larger in comparison to those in Fig. 7.

**Fig. 9:**
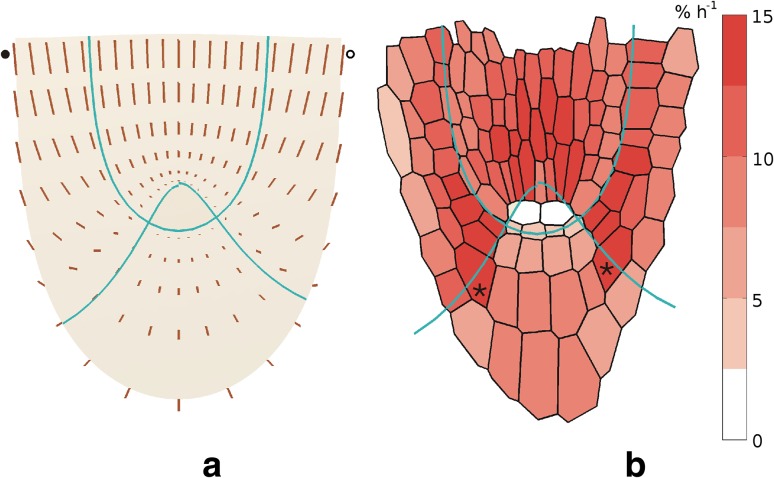
The velocity field (**a**) and distribution of the volumetric growth rates (**b**) for the *A. thaliana* root apex obtained as in Fig. 6 but taking asymmetry of the columella cell files into account. The border between the central and lateral root cap with epidermis is described by *u*_0_ = 0.27 for the *left*, whereas *u*_*0*_ = 0.35 for the *right* side of the root apex. Values of the marked vectors are (μm min^−1^): 0.102 (*open circle*) and 0.093 (*closed circle*), for the cells marked by asterisks-see text

In all cases, so far **V** field was symmetrical with respect to the root axis. However, looking at Fig. 1, some asymmetry of cell pattern can be seen, especially in distribution of the columella cell files on both sides of the root axis. In terms of the present approach, such asymmetry may be taken into account assuming different values of *u*_0_ as borders between the central and the lateral root cap with epidermis on both sides of the root axis. The **V** field and predicted distribution of the volumetric growth rate generated under *u*_0_ = 0.27 for the left, and *u*_0_ = 0.35 for the right sides, are shown in Fig. 9. In contrast to Fig. 6, now the **V** field and distribution of the volumetric growth rate are asymmetrical. Such asymmetry is evident in Fig. 9a, lengths of **V** vectors on the left side are larger than those on the right and additionally some changes in orientation of the vectors occur, especially in apical region of the lateral root cap and epidermis. Comparing vectors attached in corresponding points of this zone on both sides of the root axis (Fig. 9a), those in basal part have the same orientation but differ in length whereas those in the apical part differ less in length but also in orientation. In Fig. 9b showing the growth rate distribution the asymmetry is less pronounced than in the case of **V** field, because the changes in *R*_vol_ values are too small to go beyond the fixed ranges of the colour coding. Moreover, at first glance, the rate distribution seems to be less asymmetrical than those obtained earlier in Fig. 5b, note that values of the rate for two cells indicated by asterisks previously clearly different, here are more or less similar. We will return to that problem in the discussion.

## Discussion

This paper presents the method of determination of the displacement velocity field for the root apex. The method, combining mathematical modelling based on the continuity equation and fragmentary empirical data about **V**, can be used to calculate velocities for any region of any root apex. Here, using *A. thaliana* root as the example, it has been applied to determine **V** field for most apical part of this organ. The needed empirical data became the published velocity profile along the root axis above quiescent centre (Van der Weele et al. 2003) and evaluation of dimensions of cell packet is derived from the initial of epidermis and lateral root cap. The apical region, as including the initial (organizing) centre from which all tissues of the root apex derive, is especially interesting from the point of view of the root growth (Dolan et al. 1993; Barlow 1997; Jiang and Feldman 2005). However, spatial and directional variations of velocities in this region have not been demonstrated yet.

The choice of *A. thaliana* is not accidental. First, this species is considered as a model for angiosperms. Second, though general equations defining velocities in root apices were proposed almost 20 years ago (Hejnowicz and Karczewski 1993), no one has attempted to specify them for *A. thaliana*. Novelties of the present approach are the following: (1) the description of the root geometry and displacement velocity **V** in terms of the root-designated system are shown; (2) two ways of specification of components of **V** by empirical data are proposed, one uses velocity profile and the other cell packets analysis joined with computer simulations; (3) the method is developed in which values of components of **V** estimated along two exemplary lines, one running basipetally and the other acropetally can be used to determine **V** for any point of the organ; (4) the **V** field in 2D is presented in relation to distribution of the volumetric growth rates and growth-induced deformation of cell wall system; (5) the cases of **V** field modifications due to cell pattern asymmetry and changes in slope of the velocity profile are modelled.

The used equations for **V** come from Hejnowicz and Karczewski (1993). Because they were formulated for root apices in general, not taking a particular species into account, our assumptions concerning values of parameters are not the same as those applied previously. Here, smaller values of *c* and *d* are assumed (*c* = 0.8, *d* = 0.12, instead previously used *c* = 1.0, *d* = 0.3), and thus maximal velocities are lower than before, both in the root proper and the root cap. Also borders between root zones have been changed (*u*_0_ = 0.35 and 0.27 instead *u*_0_ = 0.45), and first of all taking values of *v*_0_ the same, we join the epidermis with the lateral root cap, not stele. All these differences, important in details, are a result of adaptation of the general model to the case of the *A. thaliana* root apex.

The components of **V** field were specified by empirical data. The specification of *V*_*u*_ has been done using data on the velocity profile along the root axis above the quiescent centre. Interestingly, the profile, obtained by advanced computational image analysis at high resolution (Van der Weele et al. 2003), was represented by bi-linear, and not sigmoidal function (Yin et al. 2003; Peters and Baskin 2006). In the present paper, a segment of this function corresponding to the region just above the quiescent centre has been used to specify d*u*/d*t* = *cu*. Because *V*_*u*_ depends not only on d*u*/d*t* but also *h*_*u*_; a value of this component increases with distance from the quiescent centre not exactly but almost linearly (part A of the graph in Fig. 3). However, for the root axis, the differences in comparison with Van der Welle’s data are negligible.

Other kinematic experiments, leading to **V** profile for Arabidopsis root apex, were mentioned (Basu et al. 2007; Chavarria-Krauser and Schurr 2004; Roberts et al. 2010). Their results are slightly different in comparison to those coming from Van der Weele et al. (2003), adopted here. A comparison of their results regarded as problematic (Wuyts et al. 2011) due to differing experimental methodologies and image analysis procedures. From the point of view of our studies, the difference relates to slope of the velocity profiles. Therefore, in modelling presented in the present paper, the cases of the other slope of the profile were considered (Fig. 8). The results have shown that more steep profile (*c* = 1.0) leads directly to increase, whereas more gradual (*c* = 0.6) decrease of velocities in stele and the lateral root cap with epidermis. These changes affect also orientation of **V** vectors but less and mainly in apical part of the lateral root cap and epidermis. It is worth noting that profiles with other slopes were obtained also in Van der Weele et al. (2003) studies, in particular, when roots of other species were investigated. Our modelling may be helpful to interpret difference in **V** field between these roots.

In order to specify *V*_*v*_, we measured dimensions of the cell packet originating from the initial common for the epidermis and lateral part of the root cap. The used method is similar to those demonstrated earlier on the example of shoot apices (Nakielski 1987; Hejnowicz et al. 1988). Here, nonlinearity of *V*_*v*_ (part B of the graph in Fig. 3) is more pronounced than that of *V*_*u*_. However, such deviation provided good results; both shape and size of the computer-generated cell packets have been satisfactory approximated (Figs. 4, 5). Moreover, the ratio *l*_*i*+1_/*l*_*i*_ obtained for the columella cells (Fig. 5b) decreases with increasing distance from the quiescent centre, which supports this nonlinearity. The velocities increase with distance from the quiescent centre, in the root cap at least twice slower than in the root proper, if compare points lying at similar distance from QC. However, the proportion *V*_*u*_/*V*_*v*_ relatively steeply increases and at peripheries of the lateral root cap and epidermis difference in length between the epidermal and root cap lineages may be so large as observed in Kurup et al. (2005).

The present paper assumes the absence of a rotational component of the velocity. This seems to be justified because rotation does not occur commonly in roots of the wild type (Scheres et al. 2002). Moreover, even if it occurs, kinematical effects resulting from its presence are small (Wuyts et al. 2011), and they can be neglected, especially when the most apical part of the root, up to 120 μm from a tip, is considered. For the same reason and the fact that our approach is based on the assumption of symplasticity, translations out of the axial plane, suggested as resulting from a local intrusive growth (Kidner et al. 2000), have been neglected. In general, in the present method, *V*_φ_ can be taken into account, but more empirical data for specification of this component are required. There are, for example, observations about spiralling cones formed in root cap of *A. thaliana* (Rost et al. 1996). It would be interesting to model their formation with the aid of the present method, yet today only fragmentary data on times and location of cell divisions that lead to such spiralling cones formation are available.

The **V** field was generated for symmetry and asymmetry cases (Figs. 6a, 9a). In the root apex with the cell pattern shown in Fig. 1, the asymmetrical field is suggested to occur. First, there is asymmetrical distribution of the columella cell files on both sides of the root axis. Second, corresponding cell packets that develop in stele and the lateral root cap with epidermis on opposite sides of the apex show difference in the longitudinal dimension, namely those on the left side are larger than those on the right (as the packets corresponding to those originated from the initials *i*_1_, *i*_4_). Some asymmetry, but rather not so evident like here, may result from random difference in division events in corresponding cells. However, more probable is difference in velocities on both sides of the root axis, as that the velocity increases proportionally to distance from the quiescent centre, and such distance for *u*_0_ = 0.27 (left side) is greater than for *u*_0_ = 0.35 (right side).

For symmetrical and asymmetrical **V** fields, surprisingly the former gave less asymmetrical distribution of the volumetric growth rate, than the latter (Figs. 6, 9). This is because in the *R*_vol_ maps two overlapping effects are manifested, one (primary) resulting from symmetry/asymmetry of **V** field and the other (secondary) resulting from asymmetry of the cell pattern in the root apex. In the case of asymmetrical **V** field (Fig. 9b), the primary effect leads to diversification, whereas the secondary effect to unification of *R*_vol_ distribution on both sides of the root axis, due to more realistic description of the asymmetrical cell pattern. The latter is simply dominating.

The present method demonstrates how to calculate velocities in the most apical part of the root apex, otherwise difficult to obtain. It combines modelling and different empirical data, but the results are visualized using a single root apex, as the example. Furthermore, the velocity profile used to specify *V*_*u*_ comes from one study and the cell pattern with cell packets used to specify *V*_*v*_, from another. Also in order to show the modelling in a way as simple as possible, we consider single but most suitable cell packets. We accept such “junction” since it demonstrates how to model **V** field and what types of empirical data are required for it. Every modelling needs strong and reliable, but not necessarily own and numerous empirical data. We have our own micrographs of Arabidopsis root apex, yet we decided to work with commonly known cell pattern regarded as typical for this species. Such typical cell pattern was necessary to illustrate ‘on cells’ both our assumption and results. Otherwise, it would be impossible to demonstrate how the coordinate system is applied to the root apex (Fig. 1), and what different growth-resulted deformations (Figs. 7, 8) occur in cells located in particular parts of the apex. In addition, asymmetry of the cell pattern was valued. The application of the method to analyze the effect of the cell pattern asymmetry on **V** field is unique. Obviously, more detailed studies, in particular, dealing with roots at especially interesting developmental stages or comparing **V** field in roots at different ages, need to be based on larger number of images and comparable data. Note that even in the present paper, in order to verify the value of the parameter *d* (that specified *V*_*v*_), other data pertaining to the same cell packets, but coming from other published roots apices, have been taken into an account.

The used modelling offers a possibility to determine **V** in the organ as the whole, if fragmentary empirical data about growth are available. Here, such data came from the velocity profile along the root axis above the quiescent centre and cell packets in the root cap. However, they could come, for example, from root live-cell lineages marked genetically like those demonstrated by Kurup et al. (2005) for the epidermis and lateral root cap (in terms of our approach corresponding to zone 4). The **V** field obtained in this way may be useful to analyze clonal relationships for any cell lineage in the whole root apex including regions difficult for empirical (even molecular) exploration. In the present paper, steady root growth was considered. It is known that during such growth, initial cells are more or less permanent, whereas, for example, as a result of laser ablation of the quiescent centre they are redefined and cell fates as well as signaling change (Van den Berg et al. 1995). Is it possible to interpret this phenomenon in a broader context without knowledge what happens with **V** field? For such interpretation, our method with modelling working close to empirical data can be helpful.

The symplastic growth has a tensor nature (Silk and Erickson 1979; Hejnowicz and Romberger 1984) which is manifested in the property that the field of growth rate of the organ is of a tensor type and at every point unless growth is isotropic, three mutually orthogonal principal growth directions can be recognized. These principal directions are postulated to define orientation of cell divisions (Hejnowicz 1984, 1989) affecting cell pattern of the growing organ. Their role in plant morphogenesis can be studied using the second rank operator called the growth tensor (Hejnowicz and Romberger 1984), calculated as gradient of **V**. Having **V** field specified in the present paper, tensor field of growth rate for the *A. thaliana* root apex has been determined (Nakielski and Lipowczan, unpublished). Analysis of such field focused on spatial and directional variation of the linear growth rates within this organ is currently prepared. It gives a possibility to analyze anisotropy of growth rate in cell walls at any position within the root apex.

## Electronic supplementary material

Below is the link to the electronic supplementary material.

Supplementary material 1 (PDF 130 kb)

Supplementary material 2 (PDF 326 kb)

## Abbreviations

*R*_vol_: Elemental rate of the volumetric growth
RC: Root-designated coordinates
3D: Three dimensional
QC: Quiescent centre
