# Supplementary material for: A method to determine the displacement velocity field in the apical region of the Arabidopsis root
Source: Planta. 2012 Jul 25;236(5):1547–57. doi: 10.1007/s00425-012-1707-x (PMC3481058; doi:10.1007/s00425-012-1707-x)
Supplement: Supplementary file 1 — Supplementary material 1 (PDF 130 kb) [file 425_2012_1707_MOESM1_ESM.pdf]

A method to determine the displacement velocity field in the apical region of the Arabidopsis root

Jerzy Nakielski, Marcin Lipowczan

<u>Online Resource S1:</u> The curvilinear orthogonal coordinate system, RC(*u*,*v*,*φ*), used to describe the root apex

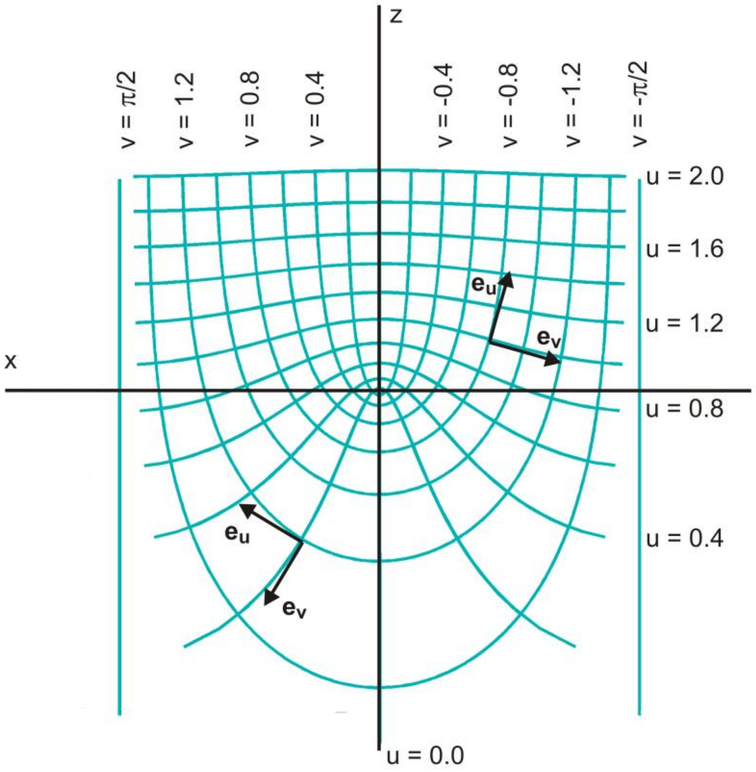

The system is represented by traces of the coordinate surfaces on the *xz* plane; $\mathbf{e}_u$ and $\mathbf{e}_v$ are two unit vectors, the third one $\mathbf{e}_\varphi$ (not shown) is perpendicular to this plane.

Coordinate equations:

$$x = \frac{2}{\pi}\arctan(\tanh u \tan v)\cos\varphi$$

$$y = \frac{2}{\pi}\arctan(\tanh u \tan v)\sin\varphi$$

$$z = \frac{1}{\pi}\log(\cosh^2 u - \sin^2 v)$$

Scale factors:

$$h_u = h_v = \frac{\sqrt{\sinh^2(2u) + \sin^2(2v)}}{\cosh^2 u - \sin^2 v}, \qquad h_\varphi = 2\arctan(\tanh u \tan v)$$
