# Supplementary material for: A method to determine the displacement velocity field in the apical region of the Arabidopsis root
Source: Planta. 2012 Jul 25;236(5):1547–57. doi: 10.1007/s00425-012-1707-x (PMC3481058; doi:10.1007/s00425-012-1707-x)
Supplement: Supplementary file 2 — Supplementary material 2 (PDF 326 kb) [file 425_2012_1707_MOESM2_ESM.pdf]

A method to determine the displacement velocity field in the apical region of the Arabidopsis root
Jerzy Nakielski, Marcin Lipowczan

<u>Online Resource S2: </u> Velocity field for the profile changed in part below QC

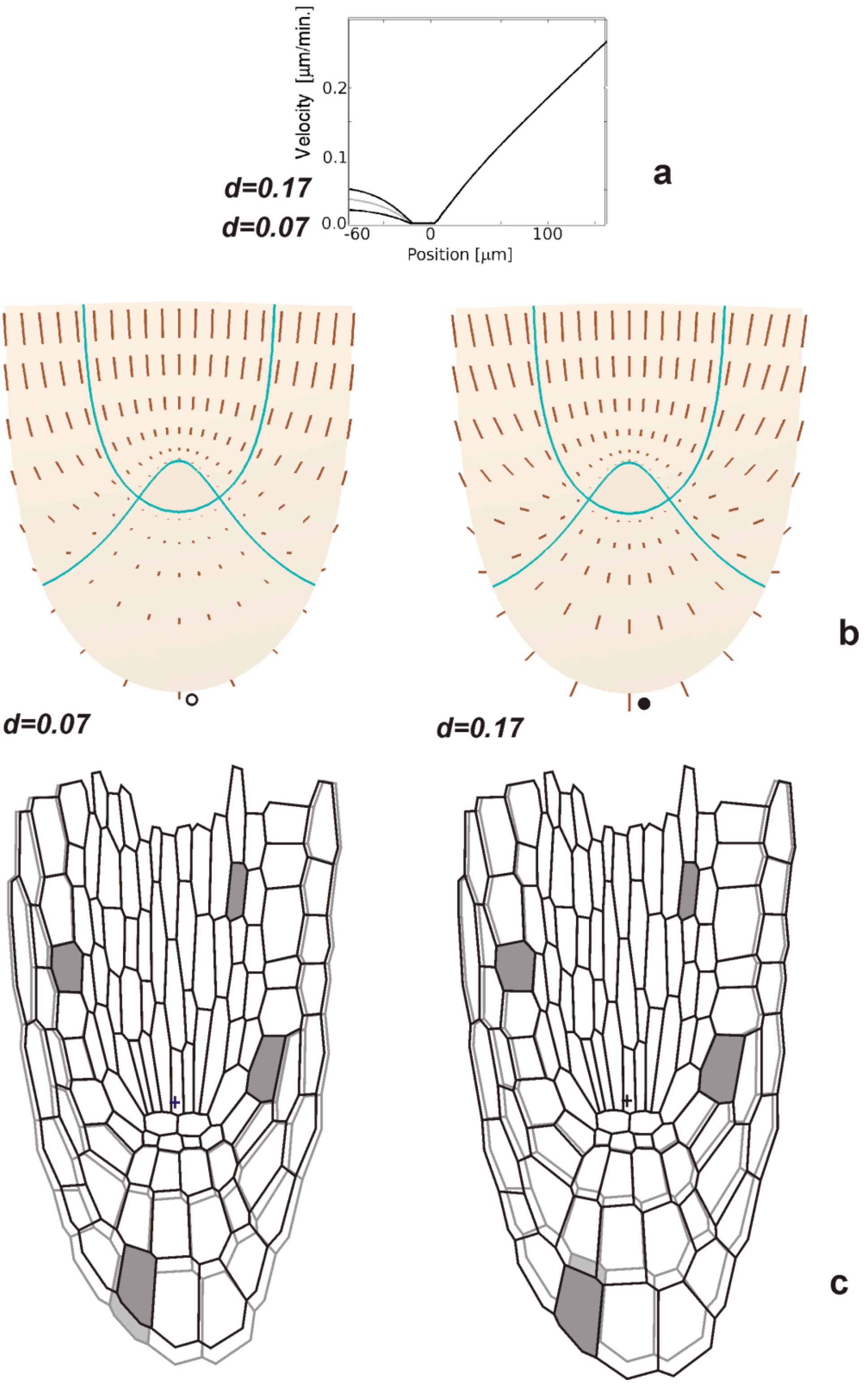

a Two velocity profiles for the root axis below QC defined by different values of the parameter *d*, in both the velocity above this zone is specified *c=0,8*; **b** V fields obtained for these profiles, values of the marked vectors in units of Fig.5a are: 0.018 μm min^{-1} (open circle) and 0.054 μm min^{-1} (closed circle); **c** the simulated cell expansion resulting from application of both fields, the results are comparable to final time-step of the sequence in Fig.6 redrawn in the background (grey).
